# Edible Herb *Aster glehni* Alleviates Inflammation and Oxidative Stress in Chondrocytes by Regulating p38 and NF-κB Signaling Pathways with Partial Involvement of Its Major Component, 3,5-Dicaffeoylqunic Acid

**DOI:** 10.3390/ijms26199691

**Published:** 2025-10-04

**Authors:** Jihyeon Baek, Hanhee Choi, Sung Ran Yoon, Yong Jin Jeong, Shin Young Oh, Min-Sook Kang, Haeng-Ran Kim, Han-Seung Shin, Seok-Seong Kang

**Affiliations:** 1 Department of Food Science and Biotechnology, College of Life Science and Biotechnology, Dongguk University, 32 Dongguk-ro, Ilsandong-gu, Goyang-si 10326, Republic of Korea; 2 Division of Agriculture Environment Research, Gyongsangbuk-do Agricultural Research and Extension Services, Daegu 41404, Republic of Korea; 3 KMF Co., Ltd., Daegu 41605, Republic of Korea; 4 Department of Food Science and Technology, Keimyung University, Daegu 42601, Republic of Korea; 5 Department of Agro-Food Resources, National Institute of Agricultural Sciences, Rural Development Administration, Wanju 55365, Republic of Korea

**Keywords:** *Aster glehni*, 3,5-dicaffeolyquinic acid, osteoarthritis, inflammation, oxidative stress

## Abstract

Osteoarthritis (OA) is primarily a degenerative disease triggered by joint inflammation and oxidative stress. While *Aster glehni* is an edible and traditionally medicinal herb, the beneficial effect of *A. glehni* on OA progression remains unknown. This study aimed to investigate the effect of *A. glehni* extract (AGE) and its primary biological compound—3,5-dicaffeoylquinic acid (3,5-DCQA)—on inflammation and oxidative stress in chondrocytes. AGE effectively inhibited the expression of interleukin (IL)-6, cyclooxygenase (COX)-2, matrix metalloproteinase (MMP)-1, and MMP-13 in chondrocytes stimulated by IL-1β for 24 h. In contrast, 3,5-DCQA did not inhibit IL-6, COX-2, and MMP expressions under the same conditions. However, when chondrocytes were stimulated by IL-1β for a short duration (6 h), 3,5-DCQA suppressed IL-6, COX-2, and MMP expressions. The inhibition of IL-6, COX-2, and MMP expressions by AGE was associated with the p38 kinase and nuclear factor-κB signaling pathways, but not ERK and JNK signaling pathways. Furthermore, AGE prevented cell apoptosis and reduced intracellular reactive oxygen species levels in chondrocytes induced by hydrogen peroxide (H_2_O_2_). AGE restored the decreased superoxide dismutase 1 and catalase mRNA expressions caused by H_2_O_2_. Collectively, AGE may protect against cartilage deterioration by inhibiting inflammation and oxidative stress, making it a promising therapeutic agent for alleviating OA.

## 1. Introduction

Osteoarthritis (OA) stands as the foremost degenerative joint disease, characterized by the degradation of cartilage and physical impairment. While the exact pathogenesis of OA remains unclear, certain risk factors are known to contribute to its development. These include age (>50 years), female sex, obesity, diabetes, and genetic predisposition [1]. However, owing to the lack of a comprehensive understanding of OA initiation and progression, effective treatment remains undeveloped. One potential pathogenesis of OA involves increased oxidative stress owing to reactive oxygen species (ROS) accumulation, possibly leading to cartilage damage and OA progression [2]. Moreover, inflammation plays a direct role in OA progression [3].

OA triggers chronic low-grade inflammation in the synovial fluids, stimulating chondrocytes to produce pro-inflammatory cytokines and enzymes that degrade cartilage [4]. Pro-inflammatory cytokines, such as interleukin-6 (IL-6), along with inflammatory mediators such as nitric oxide (NO) and cyclooxygenase-2 (COX-2), exacerbate inflammation in the articular cartilage of OA [5,6]. Cartilage degradation significantly occurs owing to the release of matrix metalloproteinases (MMPs) induced by NO. Moreover, COX-2-mediated prostaglandin E2 inhibits extracellular matrix synthesis, exacerbating cartilage degradation [7].

*Aster glehni* (AG) is an edible plant and a medicinal herb to alleviate cough, fever, pain, and insomnia in Korea [8]. Additionally, ethanol extract of AG (AGE) has exhibited anti-inflammatory properties in colonic tissues [9]. AGE similarly demonstrates the ability to suppress inflammatory mediator production by inhibiting the activation of nuclear factor-κB (NF-κB) in lipopolysaccharide (LPS)-induced microglial cells [10]. A high-performance liquid chromatography analysis was conducted [11], revealing that AG primarily contains six caffeoylquinic acids, with 3,5-dicaffeoylquinic acid (3,5-DCQA) being the most abundant [8]. Studies have shown the anti-inflammatory activity of 3,5-DCQA. For instance, 3,5-DCQA has been found to inhibit the production of NO in RAW264.7 cells stimulated by LPS [12]. However, the beneficial effect of AG on OA has not been revealed. Therefore, this study aimed to explore the anti-inflammatory and antioxidant effects of AGE and its major compound, 3,5-DCQA, on SW1353 human chondrocytes. This study elucidates the intracellular mechanisms contributing to the potential therapeutic effects in OA.

## 2. Results

### 2.1. AGE Suppresses IL-6 and COX-2 Expressions in Chondrocytes

**Figure 1:**
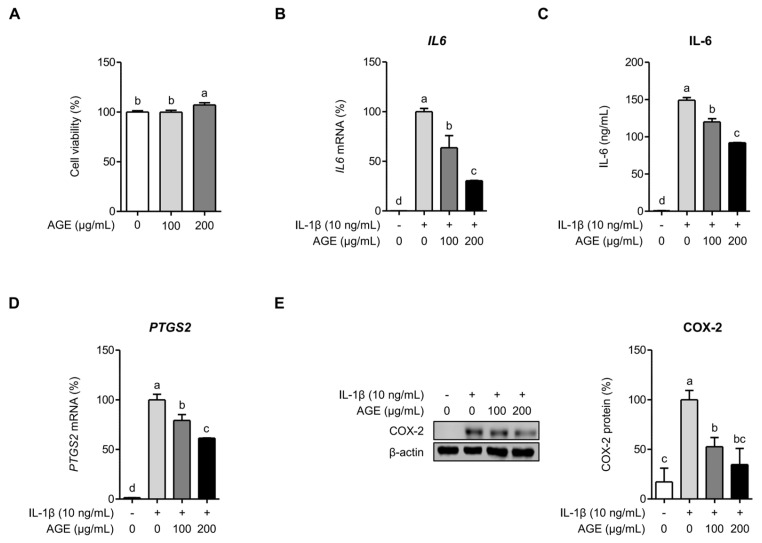
AGE inhibits IL-1β-induced inflammatory responses in chondrocytes. (**A**) SW 1353 cells were incubated with AGE (100 and 200 μg/mL) for 24 h, and cell viability was measured using the MTT assay. SW 1353 cells were pretreated with AGE (100 and 200 μg/mL) for 1 h, followed by stimulation with IL-1β (10 ng/mL) for 24 h. *IL6* (**B**) and *PTGS2* (**D**) expressions were evaluated using qRT-PCR. Protein expressions of IL-6 (**C**) and COX-2 (**E**) were assessed using ELISA and Western blot analysis, respectively. Statistical significance was determined using ANOVA (*p* < 0.05), and different letters (a–d) denote statistical significance between groups.

The effect of AGE on cytotoxicity in SW1353 cells was analyzed using the MTT (3-[4,5-dimethylthiazol-2-yl]-2,5-diphenyltetrazolium bromide) assay. Results showed that the viability of SW1353 cells remained unaffected by concentrations of 100 and 200 μg/mL of AGE (Figure 1A). Thus, these concentrations were utilized for subsequent experiments. The suppressive effect of AGE on IL-1β-induced inflammatory responses was examined by assessing the mRNA and protein levels of IL-6 and COX-2 in SW1353 cells. IL-6 mRNA and protein levels increased via IL-1β stimulation; however, they were significantly inhibited by AGE pretreatment in SW1353 cells (Figure 1B,C). IL-6 mRNA (*IL6*) expression was reduced by 36% and 70% following pretreatment with AGE at concentrations of 100 and 200 μg/mL of AGE, respectively (*p* < 0.05) (Figure 1B). IL-6 protein concentration decreased by 20% and 38% following pretreatment with AGE at concentrations of 100 and 200 μg/mL, respectively (*p* < 0.05) (Figure 1C). Additionally, AGE significantly reduced the COX-2 mRNA and protein expression, which had been increased via IL-1β stimulation in SW1353 cells. COX-2 mRNA (*PTGS2*) expression decreased by 21% and 39% following pretreatment with 100 and 200 μg/mL of AGE, respectively (*p* < 0.05) (Figure 1D). COX-2 protein levels exhibited reductions of 47% and 65% at AGE pretreatment concentrations of 100 and 200 μg/mL, respectively (*p* < 0.05) (Figure 1E).

### 2.2. AGE Inhibits MMP Expressions but Enhances Type II Collagen and Aggrecan Expressions in Chondrocytes

**Figure 2:**
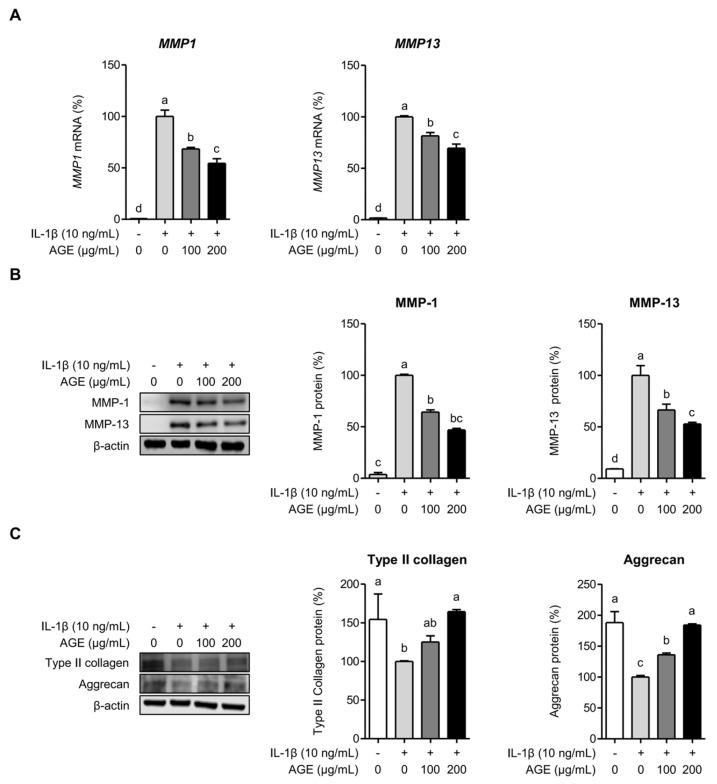
AGE inhibits IL-1β-induced MMPs, type II collagen, and aggrecan in chondrocytes. SW1353 cells were pretreated with AGE (100 and 200 μg/mL) for 1 h and then stimulated with IL-1β (10 ng/mL) for 24 h. (**A**) *MMP1* and *MMP13* expressions were assessed using qRT-PCR. Protein expressions of MMP-1, MMP-13 (**B**), type II collagen, and aggrecan (**C**) were assessed using Western blot analysis. Statistical significance was determined using ANOVA (*p* < 0.05), and different letters (a–d) denote statistical significance between groups.

To investigate whether AGE suppressed MMP expressions induced by IL-1β in SW1353 cells, we assessed the mRNA and protein expressions of MMP-1 and MMP-13. The mRNA expressions of MMP-1 (*MMP1*) and MMP-13 (*MMP13*) were significantly upregulated in SW1353 cells following exposure to IL-1β for 24 h. However, pretreatment with AGE exhibited a dose-dependent reduction in the mRNA levels of these MMPs. *MMP1* expression decreased by 32% and 46% at concentrations of 100 and 200 μg/mL of AGE, respectively (*p* < 0.05), while *MMP13* expression was attenuated by 19% and 30% at the corresponding AGE concentrations (*p* < 0.05) (Figure 2A). SW1353 cells significantly produced MMP-1 and MMP-13 proteins following stimulation with IL-1β for 24 h, while AGE pretreatment demonstrated a dose-dependent reduction in the production of MMP-1 and MMP-13 proteins. The protein level of MMP-1 decreased by 36% and 53% at 100 and 200 μg/mL concentrations of AGE, respectively, in SW1353 cells induced by IL-1β for 24 h (*p* < 0.05). The protein levels of MMP-13 were reduced by 34% and 47% at the corresponding AGE concentrations (*p* < 0.05) (Figure 2B). Furthermore, we evaluated the expressions of type II collagen and aggrecan—essential components of the cartilage matrix. Figure 2C shows that IL-1β significantly reduced the production of type II collagen and aggrecan. However, AGE pretreatment dose-dependently restored the production of type II collagen and aggrecan, which had been suppressed by IL-1β (*p* < 0.05). These findings suggest that AGE may mitigate the degradation of the cartilage matrix by downregulating MMP expression and increasing type II collagen and aggrecan production.

### 2.3. 3,5-DCQA Is Partly Involved in the Suppression of IL-1β-Induced Inflammatory Mediators and MMPs in Chondrocytes

**Figure 3:**
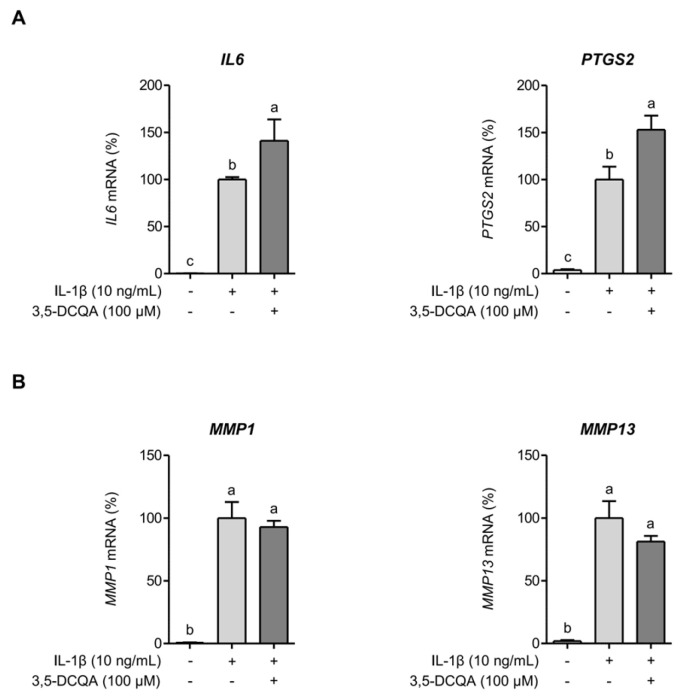
3,5-DCQA does not inhibit *IL6*, *PTGS2*, *MMP1*, and *MMP13* expressions in SW1353 cells stimulated by IL-1β for 24 h. SW1353 cells were pretreated with 3,5-DCQA (100 μM) for 1 h and then stimulated with IL-1β (10 ng/mL) for 24 h. Subsequently, *IL6*, *PTGS2* (**A**), *MMP1*, and *MMP13* expressions (**B**) were assessed using qRT-PCR. Statistical significance was determined using ANOVA (*p* < 0.05), and different letters (a–c) denote statistical significance between groups.

**Figure 4:**
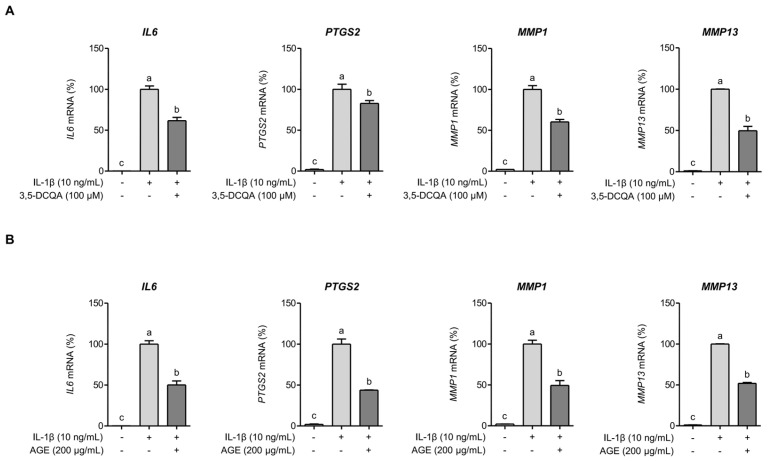
3,5-DCQA inhibits *IL6*, *PTGS2*, *MMP1*, and *MMP13* expressions in SW1353 cells stimulated by IL-1β for 6 h. SW1353 cells were pretreated with 3,5-DCQA (100 μM) (**A**) or AGE (200 μg/mL) (**B**) for 1 h and then stimulated with IL-1β (10 ng/mL) for 6 h. Subsequently, *IL6*, *PTGS2*, *MMP1*, and *MMP13* expressions were assessed using qRT-PCR. Statistical significance was determined using ANOVA (*p* < 0.05), and different letters (a–c) denote statistical significance between groups.

A previous report identified 3,5-DCQA as the predominant phytochemical in AGE [8]. Our analysis also confirmed that 3,5-DCQA is an abundant constituent of AGE (Figure S1) and several studies have shown its anti-inflammatory activity [12,13]. However, our findings indicate that 3,5-DCQA did not suppress the expressions of *IL6* and *PTGS2* in SW1353 cells when exposed to IL-1β for 24 h (Figure 3A). The mRNA expressions of *MMP1* and *MMP13* appeared to decrease slightly in SW1353 cells under comparable condition, but not statistically significant (Figure 3B). These findings suggest that 3,5-DCQA alone does not directly contribute to the suppression of IL-1β-stimulated inflammatory responses and MMP expressions during prolonged exposure. However, upon exposing SW1353 cells to IL-1β for a shorter period (6 h) instead of the previously described 24 h exposure, 3,5-DCQA significantly inhibited the expressions of *IL6*, *PTGS2*, *MMP1*, and *MMP13* (*p* < 0.05) (Figure 4A). SW1353 cells were pretreated with AGE and subsequently exposed to IL-1β for 6 h to evaluate the suppressive effect of 3,5-DCQA. AGE significantly inhibited the expressions of *IL6*, *PTGS2*, *MMP1*, and *MMP13* (*p* < 0.05) (Figure 4B). These findings indicate that during short-term exposure to IL-β, 3,5-DCQA exhibits clear inhibitory effects on the expressions of inflammatory mediators and MMPs in SW1353 cells. However, these inhibitory effects diminish with prolonged exposure to IL-1β, indicating a time-dependent and partial involvement in suppressing IL-1β-induced responses. Therefore, unlike AGE, 3,5-DCQA does not provide sustained suppression of inflammatory mediators and MMPs, suggesting that it contributes only partially to the overall osteoprotective and chondroprotective effects of AGE.

### 2.4. AGE Inhibits IL-1β-Induced Expressions of IL-6, COX-2, and MMPs Through the Suppression of p38 Kinase and NF-κB Signaling Pathways

**Figure 5:**
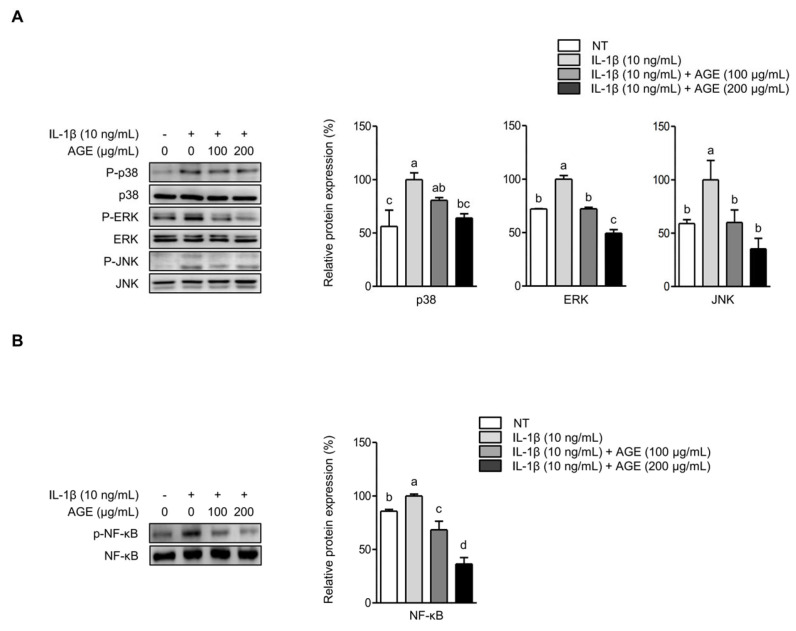
AGE inhibits IL-1β-induced phosphorylation of MAPKs and NF-κB in chondrocytes. SW1353 cells were pretreated with AGE (100 and 200 μg/mL) for 1 h and then stimulated with IL-1β for 24 h. Phosphorylated MAPKs (p38, ERK, and JNK) (**A**) and NF-κB (**B**) were assessed using Western blot analysis. The relative expressions of phosphorylated p38, ERK, JNK, and NF-κB are presented as the mean ± standard deviation. Statistical significance was determined using ANOVA (*p* < 0.05), and different letters (a–d) denote statistical significance between groups.

**Figure 6:**
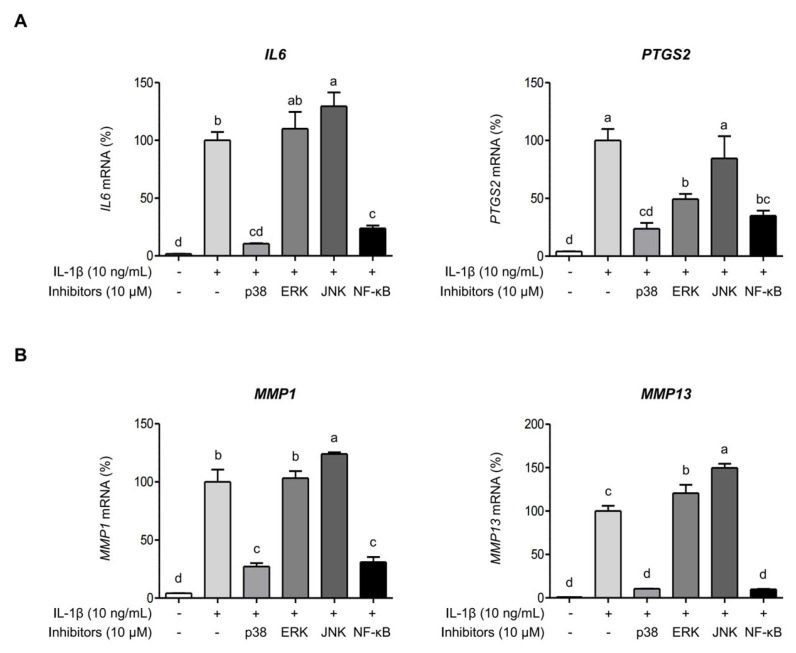
IL-1β induces *IL6*, *PTGS2*, *MMP1*, and *MMP13* expressions via p38 and NF-κB signaling pathways. SW1353 cells were pretreated with 10 μM specific inhibitors of p38, ERK, JNK, or NF-κB for 1 h and then stimulated with IL-1β for 24 h. Subsequently, *IL6*, *PTGS2*, (**A**) *MMP1*, and *MMP13* expressions (**B**) were evaluated using qRT-PCR. Statistical significance was determined using ANOVA (*p* < 0.05), and different letters (a–d) denote statistical significance between groups.

The signaling pathways of MAPKs and NF-κB are closely associated with OA progression [14]. Hence, we examined whether AGE pretreatment could reduce the expressions of IL-6, COX-2, and MMPs by influencing the signaling pathways of MAPKs and NF-κB. Figure 5A illustrates that IL-1β stimulation significantly elevated the phosphorylation of MAPKs, such as p38 kinase, ERK, and JNK, in SW1353 cells. However, AGE pretreatment significantly decreased the phosphorylation of p38 kinase, ERK, and JNK. Moreover, IL-1β led to increased phosphorylation of NF-κB, while AGE pretreatment significantly reduced NF-κB phosphorylation in SW1353 cells (Figure 5B). Thus, these findings suggest that AGE mitigates the expressions of IL-6, COX-2, and MMPs by suppressing MAPK and NF-κB signaling pathways. SW 1353 cells were stimulated with IL-1β in the presence of specific inhibitors for p38 kinase, ERK, JNK, or NF-κB to validate the role of MAPKs and NF-κB in eliciting IL-6, COX-2, MMP-1, and MMP-13 expression in response to IL-1β. Figure 6A shows that IL-1β significantly increased *IL6* and *PTGS2* expressions (*p* < 0.05). The presence of p38 kinase- and NF-κB-specific inhibitors significantly reduced *IL6* and *PTGS2* expressions, while ERK- and JNK-specific inhibitors did not decrease IL-1β-induced *IL6* and *PTGS2* expressions. Similarly, IL-1β significantly elevated *MMP1* and *MMP13* expressions. However, the inhibition of p38 kinase and NF-κB signaling resulted in a significant reduction in *MMP1* and *MMP13* expressions, while the inhibition of ERK and JNK did not decrease these mRNA expressions (Figure 6B). These findings suggest that the expressions of IL-6, COX-2, and MMPs induced by IL-1β are dependent on the signaling pathways of p38 kinase and NF-κB. While AGE effectively suppressed the signaling transduction of all MAPKs, p38 kinase, ERK, JNK, and NF-κB, only p38 kinase and NF-κB were implicated in AGE-mediated inhibition of inflammatory mediators and MMP expression.

### 2.5. AGE Attenuates Hydrogen Peroxide (H_2_O_2_)-Induced Oxidative Stress in Chondrocytes

**Figure 7:**
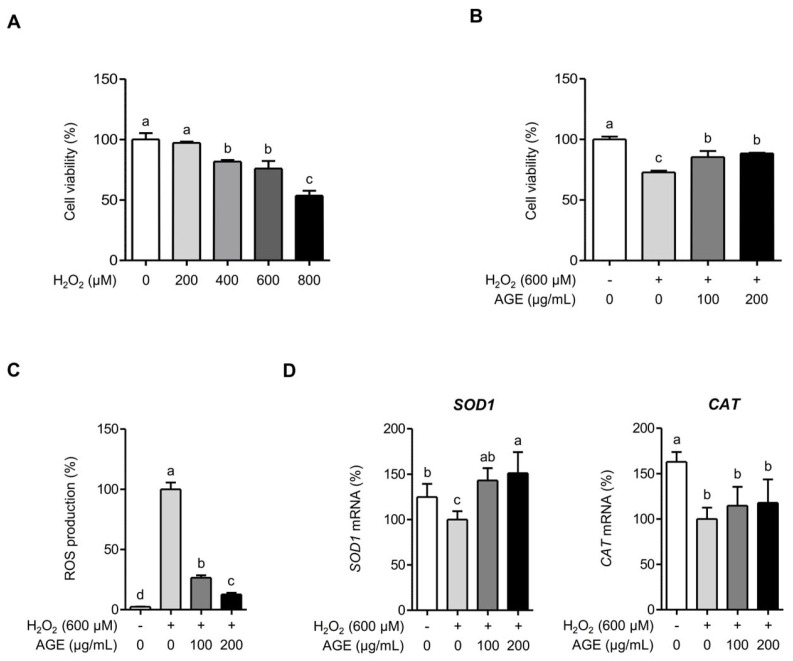
AGE exhibits antioxidant activities against H_2_O_2_-induced oxidative stress in chondrocytes. (**A**) SW1353 cells were exposed to varying concentrations of H_2_O_2_ (200, 400, 600, and 800 μM) for 24 h, followed by an assessment of the cell viability using an MTT assay. (**B**) SW1353 cells were pretreated with varying concentrations of AGE (100 and 200 μg/mL) for 1 h before stimulation with H_2_O_2_ (600 μM) for 24 h. Cell viability was determined using an MTT assay. (**C**) SW1353 cells were pretreated with AGE (100 and 200 μg/mL) and stimulated with H_2_O_2_ (600 μM) for 6 h. Subsequently, ROS production was measured using a ROS assay kit. (**D**) SW1353 cells were pretreated with AGE (100 and 200 μg/mL) and stimulated with H_2_O_2_ (600 μM) for 6 h. Following this, *SOD1* and *CAT* expressions were assessed using qRT-PCR. Statistical significance was determined using ANOVA (*p* < 0.05) and different letters (a–d) indicating statistical significance between groups.

H_2_O_2_ has been commonly used to induce oxidative stress in different cell types, such as chondrocytes, due to its role as a primary source of endogenous ROS [15]. To explore the effect of H_2_O_2_ on SW 1353 cells, cell viability was assessed using an MTT assay. Treatment with H_2_O_2_ at different concentrations (200, 400, 600, and 800 μM) led to a significant decrease in cell viability at 400, 600, and 800 μM, but not at 200 μM (*p* < 0.05) (Figure 7A). To assess the effect of AGE on H_2_O_2_-induced decrease in cell viability, SW 1353 cells were pretreated with AGE for 1 h followed by stimulation with 600 μM H_2_O_2_. Figure 7B illustrates that AGE effectively prevented the decrease in the cell viability induced by H_2_O_2_, suggesting its potential protective effect against H_2_O_2_-induced apoptosis. As shown in Figure 7C, H_2_O_2_ significantly elevated intracellular ROS levels; however, in SW1353 cells, the intracellular ROS levels were significantly reduced by 73% and 87% at the respective concentrations of 100 and 200 μg/mL of AGE. To investigate whether AGE upregulates the expression of superoxide dismutase 1 (*SOD1*) and catalase (*CAT*), which are crucial antioxidant enzymes, we examined their mRNA expression in the presence or absence of AGE. H_2_O_2_ reduced the mRNA expressions of *SOD1* and *CAT*. However, AGE pretreatment significantly increased *SOD1* expression (*p* < 0.05). While the rise in *CAT* expression with AGE pretreatment was not statistically significant, a slight upregulation was observed (Figure 7D).

## 3. Discussion

Various edible plants and their active compounds exhibit osteoprotective and chondroprotective properties. They achieve this by suppressing the production of inflammatory mediators, such as IL-6 and COX-2, as well as matrix-degrading enzymes, such as MMP-1 and MMP-13. These inhibitory effects occur from the regulation of MAPKs and NF-κB signaling pathways induced by IL-1β [16]. However, our study explores the preventive effects of AGE on IL-1β-treated chondrocytes through the suppression of inflammatory responses, matrix-degrading enzymes and oxidative stress. We found that AGE pretreatment mitigates inflammatory responses and the degradation of the cartilage matrix by regulating the expression of MMPs, type II collagen, and aggrecan. Additionally, AGE protects the cells from oxidative stress, a factor closely linked to OA development [17]. While our findings suggest the potential of AG in mitigating OA, the most prevalent biological compound in AGE, 3,5-DCQA, appears nonessential for this mitigation. However, in contrast to this study, studies have shown the anti-inflammatory effects of 3,5-DCQA. For instance, 3,5-DCQA inhibited nitric oxide production in RAW 264.7 cells when exposed to *Escherichia coli* LPS [12]. In addition, we revealed the anti-inflammatory activity of 3,5-DCQA in RAW 264.7 cells stimulated by *Porphyromonas gingivalis* LPS [18]. Although 3,5-DCQA is one of the major components in AGE, our findings indicated that its effects on IL-1β-induced inflammatory responses were minimal compared with those of the whole AGE. Future studies involving fractionation of AGE and systematic evaluation of 3,5-DCQA as well as other components will be essential to clarify their specific roles in the observed protective effects.

Increasing evidence indicates that inflammation plays a significant role in OA pathology [19]. Pro-inflammatory cytokines, such as IL-1β, are pivotal in OA pathophysiology, reducing cartilage anabolism and encouraging cartilage catabolism [14]. Thus, IL-1β is commonly used to stimulate chondrocyte models for understanding OA pathogenesis. IL-1β also stimulates the production of IL-6, which actively contributes to OA progression [20]. A previous study showed that COX-2 can stimulate MMP production, potentially resulting in the degradation of the cartilage matrix [21]. Consequently, therapeutic strategies have prioritized reducing inflammatory responses in OA treatment. Here, AGE effectively reduced IL-6 and COX-2 production in chondrocytes exposed to IL-1β. This suggests that reducing inflammatory responses may decrease MMP production, thus preserving cartilage matrix integrity. The excessive production of MMPs exacerbates OA progression [22]. Type II collagen and aggrecan are key components of cartilage proteins crucially involved in synthesizing matrix-related proteins in cartilage [14,17]. Abnormal expression of MMPs inhibits the synthesis of type II collagen and aggrecan, leading to proteolysis and pathological cartilage breakdown in OA [23]. In this study, IL-1β-stimulated SW1353 cells show increased expression of MMP-1 and MMP-13, alongside significant reductions in matrix-related proteins, type II collagen, and aggrecan.

MAPK signaling pathways, which include p38, ERK, and JNK pathways, play a crucial role in regulating cellular proliferation, differentiation, apoptosis, and inflammatory responses in mammalian cells [24]. MAPK signaling pathways also regulate the expression of MMPs, such as MMP-13 [25]. Additionally, activation of the NF-κB pathway regulates the production of inflammatory mediators and MMPs during OA development [14]. Studies have shown that MAPK signaling pathways contribute to the production of aggrecanases and MMPs, which are linked to cartilage matrix degradation [26]. Activation of the NF-κB pathway accelerates the expression of MMPs and inflammatory mediators, such as COX-2, resulting in adverse effects on joint health [27]. Therefore, targeting MAPK and NF-κB signaling is essential to preserve joint health. Our findings suggest that AGE pretreatment effectively disrupts the signal transduction of MAPKs and NF-κB, indicating that the protective effect of AGE is associated with the inhibition of MAPKs and NF-κB signaling. More specifically, we revealed only p38 kinase among the MAPKs and NF-κB signaling could be critically important to attenuate OA progression by AGE pretreatment.

Oxidative stress plays a role in OA progression by damaging the cartilage matrix and triggering inflammation [28]. Additionally, it induces chondrocyte apoptosis, which contributes to cartilage matrix degradation in OA [29]. Our study showed that the presence of H_2_O_2_, which is commonly used to induce oxidative stress, significantly decreases the viability of SW1353 cells. However, cell viability was restored in the presence of AGE, suggesting that AGE suppresses cellular apoptosis. Studies show that excessive production of ROS contributes to OA progression, resulting in heightened inflammation [3,28]. Although we did not directly assess the expression of inflammatory mediators in H_2_O_2_-stimulated SW 1353 cells or the anti-inflammatory activity of AGE, AGE is assumed to mitigate oxidative stress, thereby reducing inflammatory responses. Furthermore, oxidative stress and inflammation in chondrocytes are closely linked to OA severity and development [28]. Hence, our findings suggest that AGE might target the reduction of oxidative stress and inflammation, ultimately providing protection against OA.

## 4. Materials and Methods

### 4.1. AGE Preparation

AGE was kindly provided by Prof. Han-Seung Shin at Department of Food Science and Biotechnology, Dongguk University (Seoul, Republic of Korea). Briefly, AG leaves were homogenized using a blender and sonicated with a sonicator (Sonic & Materials, Inc., Newtown, CT, USA). The homogenized material was then refluxed with 70% ethanol at 80 °C for 6 h. The extract was filtered, evaporated, and freeze-dried. The freeze-dried powder was resuspended in phosphate-buffered saline (PBS) and stored at −80 °C until subsequent use.

### 4.2. Cell Culture

A human chondrosarcoma cell line SW1353 was purchased from American Type Culture Collection (Manassas, VA, USA) and cultured in Dulbecco’s Modified Eagle’s Medium (DMEM; Welgene, Gyeongsan, Republic of Korea) supplemented with 10% fetal bovine serum (Gibco, Burlington, ON, Canada), 100 U/mL penicillin and 100 μg/mL streptomycin (HyClone, Logan, UT, USA) in a humidified atmosphere of 5% CO_2_ at 37 °C.

### 4.3. Quantitative Reverse Transcription Polymerase Chain Reaction (qRT-PCR)

SW1353 cells were seeded in a 6-well plate at a density of 2.5 × 10^5^ cells/mL and incubated in a complete DMEM medium at 37 °C for 24 h to analyze expressions of *IL6*, *PTGS2*, *MMP1*, and *MMP13*. The cells were pretreated with AGE (100 or 200 μg/mL) or 3,5-DCQA (100 μM; Sigma-Aldrich, St. Louis, MO, USA) for 1 h. Subsequently, the cells were stimulated with 10 ng/mL IL-1β (R&D Systems, Minneapolis, MN, USA) at 37 °C for either 6 or 24 h. The cells were pretreated for 1 h because this interval showed the most effective suppression of inflammatory responses in preliminary tests. In a separate experiment, SW1353 cells were seeded and cultured as described above. Following that, the cells underwent pretreatment with AGE (100 or 200 μg/mL) for 1 h and were stimulated with H_2_O_2_ at 37 °C for 6 h to assess the expressions of *SOD1* and *CAT*. Following stimulation, total RNA was extracted from the cells using a TRIzol reagent (Invitrogen, Carlsbad, CA, USA). The mixture of total RNA, random hexamers, and reverse transcriptase (Promega, Madison, WI, USA) was utilized to synthesize complementary DNA (cDNA). To assess mRNA target gene expressions, qRT-PCR was conducted. This involved amplifying target genes in a total volume of 20 μL containing cDNA, a SYBR Green Real-Time PCR master mix (Toyobo, Osaka, Japan), gene-specific primers, and sterile water using a StepOnePlus^TM^ real-time PCR system (Applied Biosystems, Foster City, CA, USA). The relative mRNA expressions were normalized to the expression of glyceraldehyde-3-phosphate dehydrogenase (*GAPDH*) through the 2^−ΔΔCt^ method. The primer sequences used for qRT-PCR are listed in Table 1.

### 4.4. Determination of IL-6 Protein

SW1353 cells were seeded in a 48-well culture plate at a density of 2.5 × 10^5^ cells/mL and incubated in complete DMEM at 37 °C for 24 h. The cells underwent pretreatment with AGE (100 or 200 μg/mL) for 1 h, followed by stimulation with IL-1β (10 ng/mL) at 37 °C for 24 h. The concentration of IL-6 protein in the culture medium was assessed using a human IL-6 enzyme-linked immunosorbent assay kit (BioLegend, San Diego, CA, USA).

### 4.5. Western Blot Analysis

SW1353 cells were initially seeded in a 6-well culture plate at a density of 2.5 × 10^5^ cells/mL and were incubated in complete DMEM at 37 °C for 24 h. Following this, the cells were pretreated with AGE (100 or 200 μg/mL) for 1 h and then stimulated with 10 ng/mL IL-1β (R&D Systems) at 37 °C for 24 h. Cell lysates were subsequently extracted using a lysis buffer, and the concentration of these lysates was assessed utilizing a bicinchoninic acid protein assay kit (Thermofisher Scientific, Waltham, MA, USA). The cell lysate, each containing an equal concentration of 30 μg, was subjected to 10% sodium dodecyl sulfate-polyacrylamide gel electrophoresis and subsequently transferred onto Immobilon^®^-Ppolyvinylidene difluoride membranes (Millipore, Bedford, MA, USA). After transfer, the membranes were blocked using 5% skimmed milk and then incubated overnight at 4 °C with specific primary antibodies. Following this, the membranes were incubated with secondary antibodies of horseradish peroxidase-conjugated anti-rabbit IgG (Cell Signaling Technology, Danvers, CA, USA) or anti-mouse IgG (Santa Cruz Biotechnology, Santa Cruz, CA, USA) at 25 °C for 2 h. Immunoreactive proteins were visualized using an enhanced chemiluminescence reagent (Dyne Bio, Seongnam, Republic of Korea) with a C-DiGit blot scanner (Li-Cor Bioscience, Lincoln, NE, USA). After acquisition on a C-DiGit blot scanner, images were analyzed in Image Studio with background subtraction. Band intensities were normalized to β-actin and expressed relative to the IL-1β-only control. All primary antibodies used for Western blot were purchased from Cell Signaling Technology.

### 4.6. Confirmation of Intracellular Signaling Pathways

To validate the involvement of mitogen-activated protein kinases (MAPKs), including p38, extracellular signal-regulated kinase (ERK), c-Jun-N-terminal kinase (JNK), and nuclear factor-κB (NF-κB) signaling pathways in the inhibitory effect of AGE on IL-1β-induced IL-6, COX-2, MMP-1, and MMP-13, SW1353 cells were pretreated with specific inhibitors: p38 (SB203580), ERK (PD98059), JNK (SP600125), and NF-κB (Bay 11-7821), each at a concentration of 10 μM for 1 h. Following this, the cells were stimulated with IL-1β (10 ng/mL) for an additional 24 h. Subsequently, total RNA was extracted from the cells, and *IL6*, *PTGS2*, *MMP1*, and *MMP13* mRNA expressions were determined as described above. All specific inhibitors were purchased from GlpBio (Montclair, CA, USA), except for PD98059, which was obtained from Sigma-Aldrich.

### 4.7. Cell Viability and Fluorometric Measurement of Reactive Oxygen Species

SW1353 cells were seeded in a 96-well culture plate at a density of 2.5 × 10^5^ cells/mL in complete DMEM at 37 °C for 24 h. The cells were subjected to treatment with H_2_O_2_ at concentrations ranging from 0 to 800 μM for 24 h. Following treatment, cell viability was assessed using an MTT assay. Briefly, MTT reagent (0.5 mg/mL) was added to each well, and the cells were incubated for an additional 3 h at 37 °C. The supernatants were removed, and dimethyl sulfoxide (200 μL/well) was added to each well. Following the dissolution of the formazan crystals, absorbance was measured at 540 nm using a microtiter plate reader (Allsheng, Hangzhou, China). All reagents utilized for the MTT assay were obtained from Duchefa Biochemie (Noord-Holland, The Netherlands). To assess the effect of AGE on the viability of SW 1353 cells in the presence of H_2_O_2_, the cells underwent pretreatment with AGE (100 and 200 μg/mL) for 1 h. Subsequently, they were stimulated with H_2_O_2_ (600 μM) for an additional 24 h. Cell viability was also determined using MTT assay, following the method described above. ROS production in SW1353 cells treated with AGE was evaluated using a cellular ROS assay kit (Abcam, Cambridge, MA, USA). SW1353 cells were seeded in a 96-well culture plate at a density of 2.5 × 10^5^ cells/mL in complete DMEM at 37 °C for 24 h. A fluorogenic dye, 2′7′-dichlorofluorescin diacetate (DCFDA), was added to each well and incubated at 37 °C for 45 min. After removing DCFDA, the cells were incubated with AGE (100 or 200 μg/mL) for 1 h, followed by treatment with 600 μM H_2_O_2_ for 6 h at 37 °C. Excitation and emission wavelengths were measured at 485 and 535 nm, respectively, using a GloMax^®^ Discover microplate reader (Promega).

### 4.8. Statistical Analysis

All results are expressed as the mean ± standard deviation. Data were analyzed using the one-way analysis of variance (ANOVA) (IBM SPSS Statistics 23 software; IBM, Armonk, NY, USA). *p* values < 0.05 were considered statistically significant.

## 5. Conclusions

In conclusion, our study is the first to explore the potential of AGE in cartilage protection by inhibiting inflammation and MMP expression through the suppression of p38 kinase and NF-κB signaling pathways. Furthermore, AGE effectively alleviates oxidative stress, contributing to the attenuation of cartilage deterioration. However, 3,5-DCQA demonstrates an inhibitory effect only in short-term exposure to IL-1β, suggesting its limited efficacy in attenuating cartilage deterioration. Consequently, AGE emerges as a promising therapeutic agent for alleviating OA.

## Figures and Tables

**Table 1:** Primer sequences for qRT-PCR analysis.

Gene	Sequence
IL-6 (*IL6*)	5′-TCCTACCCCAATTTCCAATGCT-3′ 5′-TCTGACCACAGTGAGGAATGTC-3′
COX-2 (*PTGS2*)	5′-GGCCATGGGGTGGACTTAAA-3′ 5′-CCCCACAGCAAACCGTAGAT-3′
MMP-1 (*MMP1*)	5′-AAGGCCAGTATGCACAGCTT-3′ 5′-TTTTCAACCACTGGGCCACTA-3′
MMP-13 (*MMP13*)	5′-AGACCTCCAGTTTGCAGAGC-3′ 5′-ATCAGGAACCCCGCATCTTG-3′
SOD1 (*SOD1*)	5′-AGGCATGTTGGAGACTTGGG-3′ 5′-AACGACTTCCAGCGTTTCCT-3′
CAT (*CAT*)	5′-TCTCACCAAGGTTTGGCCTC-3′ 5′-GCGGTGAGTGTCAGGATAGG-3′
GAPDH (*GAPDH*)	5′-AAGGTGAAGGTCGGAGTCAA-3′ 5′-ATGACAAGCTTCCCGTTCTC-3′

## Data Availability

Data will be made available upon reasonable request.

## Supplementary Materials

The following supporting information can be downloaded at: https://www.mdpi.com/article/10.3390/ijms26199691/s1.
