# Supplementary material for: Edible Herb Aster glehni Alleviates Inflammation and Oxidative Stress in Chondrocytes by Regulating p38 and NF-κB Signaling Pathways with Partial Involvement of Its Major Component, 3,5-Dicaffeoylqunic Acid
Source: Int J Mol Sci. 2025 Oct 4;26(19):9691. doi: 10.3390/ijms26199691 (PMC12525141; doi:10.3390/ijms26199691)
Supplement: Supplementary file 1 [file ijms-26-09691-s001.zip › ijms-3842567-supplementary.pdf]

Supplementary data

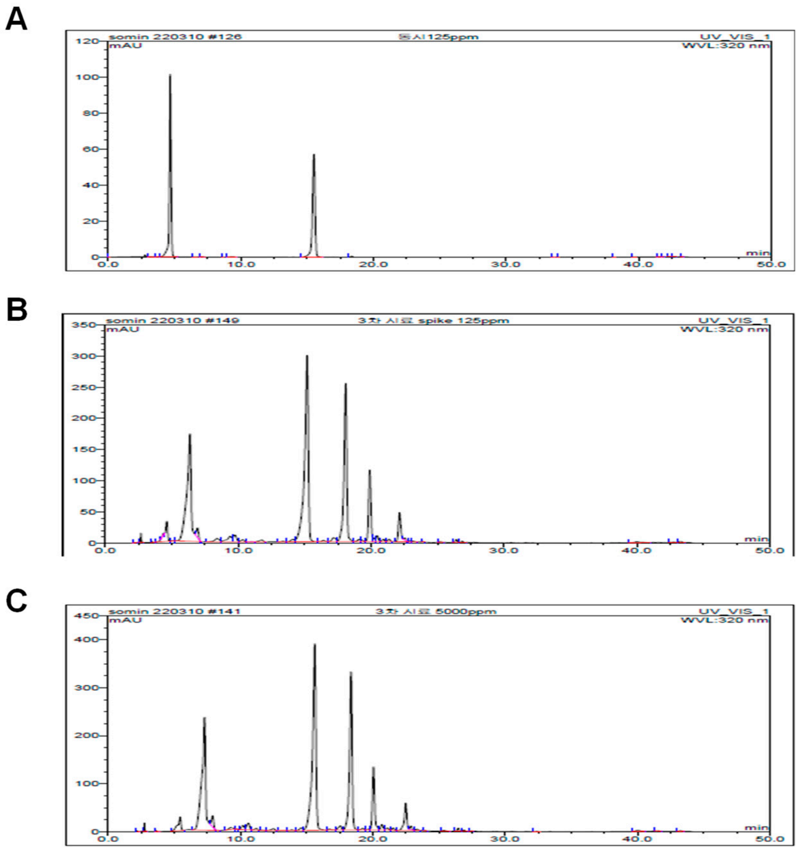

Figure S1. HPLC chromatograms of AGE showing spiked (B) and unspiked (C) samples with the standard compounds 5-caffeoylquinic acid (5-CQA) and 3,5-dicaffeoylquinic acid (3,5-DCQA) (A).
